# Supporting Active Mobility and Green Jobs through the Promotion of Cycling

**DOI:** 10.3390/ijerph14121603

**Published:** 2017-12-19

**Authors:** Rodrigo Scotini, Ian Skinner, Francesca Racioppi, Virginia Fusé, Jonas de Oliveira Bertucci, Rie Tsutsumi

**Affiliations:** 1 World Health Organization Regional Office for Europe, 2100 Copenhagen, Denmark; racioppif@who.int; 2 Transport and Environmental Policy Research, Crowborough TN6 1NE, UK; ian.skinner@tepr.co.uk; 3 United Nations Economic Commission for Europe, 1202 Geneva, Switzerland; Virginia.Fuse@unece.org; 4 Brazilian NGO Rodas da Paz, CEP 70853-040, Brasilia, Brazil; mestrejonas@gmail.com; 5 UN Environment Regional Office for Europe, 1219 Chazelaine, 1202 Geneva, Switzerland; Rie.Tsutsumi@unep.org

**Keywords:** cycling, job creation, green jobs, THE PEP, active mobility, sustainable mobility

## Abstract

This article is a summary of the main findings of the study “Riding towards the green economy: cycling and green jobs”, which was developed in the context of the Transport, Health and Environment pan-European Programme (THE PEP). It builds on previous work under THE PEP, which demonstrated the job creation potential of cycling and of green and healthy transport more generally. The report summarized in this article collected data on jobs associated with cycling directly from city authorities and analysed these to re-assess previous estimates of the job creation potential of cycling. It concluded that the number of cycling-related jobs in the pan-European Region could increase by 435,000 in selected major cities if they increased their cycling share to that of the Danish capital Copenhagen. The implications and potential role of municipal and sub-national authorities in facilitating cycling while supporting economic development are then discussed. These findings indicate that investment in policies that promote cycling could deliver not only important benefits for health, the environment and the quality of urban life, but could also contribute to a sizable creation of job opportunities. Authorities need to be proactive in promoting cycling in order to deliver these benefits.

## 1. Introduction

At the international level, there is a growing interest in active mobility policy, which is also reflected across national, regional and municipal governments. Active mobility refers in this context to mobility that includes physical activity, for example cycling and walking. Today, promoting active urban travel is an aim of growing importance to many cities in the European Region [1], especially in the European Union [2,3]. This is seen as an opportunity to improve the quality of urban life while promoting residents’ health by addressing transport-related challenges, including physical inactivity, air pollution, and congestion [4,5].

Active mobility is receiving increasing attention particularly as a highly effective means to (re)introduce or maintain physical activity in daily life [1]. Alternatives to motorized means of transportation have been shown to bring positive change beyond drops in absenteeism and improvements in health [6,7]. These include improvements to the environment, notably through reduced emissions of greenhouse gases and air pollutants, and improved quality of life, particularly in urban areas, where cycling can alleviate traffic congestion and noise, as well as reduce the need for parking spaces and for intrusive road infrastructures [8,9]. The health benefits gained by regular physical activity can be substantial (see Table 1).

This article reviews the main findings of the study “Riding towards the green economy: cycling and green jobs” [11], developed in the context of the Transport, Health and Environment Pan European Programme (THE PEP). As with previously published studies that present evidence of employment-generation from active transportation investments [12], the reviewed paper demonstrates that, in addition to its important environment and health benefits, cycling also has significant job creation potential. 

### 1.1. Evidence in Job Creation

Promoting cycling may generate three types of jobs in green and healthy transport. In order to suit the concept, these jobs need to reduce emissions and improve energy efficiency, as well as contribute to the reduction of health risks [13]. These can be direct, induced, or indirect jobs:

- Direct jobs are those involved with cycling infrastructure and maintenance and bicycle manufacture and repair related services;
- Indirect jobs are the ones in the supply chain of infrastructure maintenance and construction, bicycle manufacturing or repair industries, or in the administration of cycling systems;
- Induced jobs are created by the increased expenditures of employees working directly and indirectly in the cycling economy.

For example, increases in cycling tourism impact the wider tourist industry by generating jobs in services to cyclists, such as restaurants and hotels [13]. The box below elucidates how cycling creates jobs. Additionally, evidence indicates that monetary resources saved from substituting motor vehicle trips with active mobility and thus spent on other consumer items, tends to benefit local business activity, and generate employment [14].

### 1.2. Box 1. How Cycling Creates Jobs

Job creation linked to cycling can be initiated through public action. Investment in cycling (such as safe cycling infrastructures, support to the combined use of bicycles and public transport, bike sharing schemes) can stimulate more cycling, make it easier to choose it as a transport mode, and in turn provide the right conditions for a cycling-friendly culture. When a significant number of cyclists appear, more bicycles, more cycling equipment, maintenance and repair services are needed. As a consequence of the higher presence of bicycles in a city, a greater demand for infrastructure is generated, along with more public support for policies and public investment in cycling. Eventually, the more popular cycling becomes, the larger the incentives are for entrepreneurs to develop businesses associated with the use of bicycles and related services. Subsequently, benefits to productivity may accrue as the population becomes healthier, while reduced expenditure on transport helps to generate employment in other sectors.

### 1.3. The Transport Health and Environment Pan European Programme (THE PEP)

THE PEP was launched in 2002 to attain more sustainable and healthy patterns of transportation and mobility, and a closer incorporation of environmental and health issues into transport policies across the pan-European region.

In this context, THE PEP has placed a special emphasis on gathering, fostering and disseminating research in the field of active mobility in order to inform policy makers and promote policy change. Due to the interest of policy makers in creating jobs and reducing social inequalities, the WHO together with the United Nations Economic Commission for Europe and the United Nations Environment Programme in 2014 conducted an initial study on “Unlocking new opportunities: jobs in green and healthy transport” [13] to review the evidence for the creation of jobs in green and healthy transport. The study focused on jobs in the active modes (walking and cycling) and public transport, as these areas have been most neglected in the green jobs debate and yet appear to have a large potential in job creation [6]. 

The study summarized in this article, “Riding towards the green economy: cycling and green jobs”, [11] builds on the previous report by focusing on cycling as a means of transport and developing the evidence-base for the number of cycling jobs in cities. To the best of our knowledge, this study [11] represents the first attempt in the region to pull together evidence on the number of cycling-related jobs from cities using a standardized method.

The current article continues by setting out the methods used to estimate the number of cycling jobs (Section 2). The results are presented in Section 3, including the estimated number and, in some cases, the types of jobs created. The paper concludes with a discussion on the policy implications, the ways local governments can support job creation through cycling, the impacts on other sectors, and the gaps in research. 

## 2. Materials and Methods

**Figure 1:**
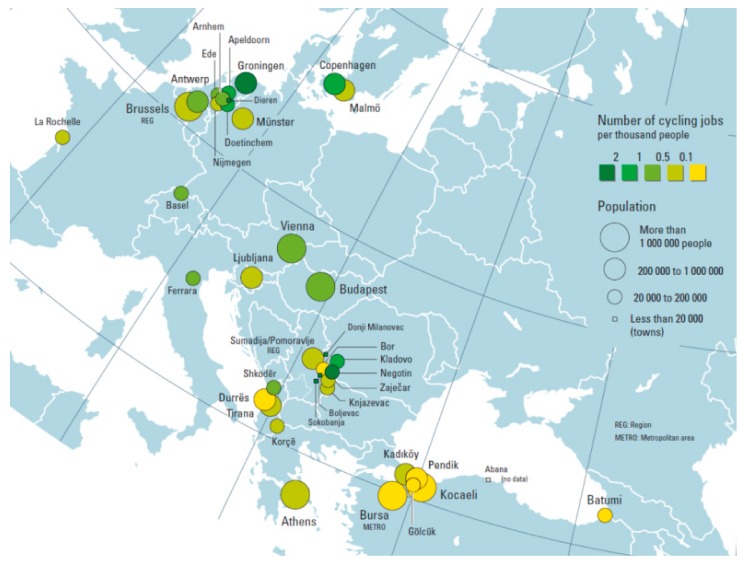
Cities and regions that supplied data for the report. Source: WHO Regional Office for Europe, United Nations Economic Commission for Europe, Riding towards the green economy: cycling and green jobs; 2016. Copenhagen: WHO EURO [11].

As already noted, the primary aim of the more recent report [11] was to gather more refined estimates on the number of cycling jobs in different cities, which could be used to complement the estimates from the previous report [13]. A survey to collect information about cycling–related jobs in cities was developed, accompanied by a guidance note. The survey was widely disseminated through city networks such as WHO’s Healthy Cities Network coordinators, UNEP’s Covenant of Mayor’s focal points, UNECE’s network of Baltic cities, ECF’s ‘Cities for Cyclists’ network, as well as POLIS and ICLEI city networks. Additionally, over 50 cities that have been reported to promote cycling were contacted directly. In this way, information and data were obtained from 37 cities in 15 countries located in the pan-European Region (see Figure 1). Generally, responses were received directly from city representatives.

The methodology used to estimate the potential increase in the number of cycling jobs was first applied in the 2014 report [13]. The estimate of the possible number of additional cycling-related jobs that might be created in the pan-European Region was based on a simple extrapolation. The basis of this was data on the cycling modal share, the associated number of cycling jobs and the population of Copenhagen, Denmark. The term modal share refers to the percentage of travelers making use of a particular mode of transportation. It was assumed that the relationship between cycling modal share and cycling jobs per capita implied by the figures for Copenhagen was constant; using this assumption, it was possible to estimate the number of existing cycling jobs in 56 major cities of the pan-European region on the basis of the cycling modal share and population of those cities. Information on the population of the cities was taken from a relevant UN database [15], while information on their cycling modal share was taken from various sources, including the website of the European Platform on Mobility Management [16] and various city-specific publications [13]. For the countries for which information on modal share was not available, low modal shares were assumed consistent with similar cities.

The estimate of the number of additional jobs that would be created if the city achieved the same modal share as Copenhagen was based on the assumption that the relationship between cycling jobs per capita and cycling modal share was linear, so that the number of jobs increased as the modal share increased. The potential number of additional cycling jobs was the difference between the “existing” number of jobs and those associated with a modal share equivalent to that of Copenhagen.

For six of the cities for which an estimate was made in the first report [13], an estimate was received directly from the city for the more recent report [11]. On average, the figures provided for the number of existing cycling-related jobs for the cities of Athens (Greece), Brussels (Belgium), Budapest (Hungary), Ljubljana (Slovenia), Tirana (Albania), and Vienna (Austria) for the second report were more than 150% higher those estimated for the first report. Further analysis demonstrated that the data on the relationship between the number of cycling related jobs per capita and the modal share of cycling derived from the previous report might also be considerably higher. With these two additional pieces of information, it was possible to make new estimates of the number of cycling-related jobs that might be created if the same 56 major cities had the same cycling modal share as Copenhagen (see Table 2).

## 3. Results

The first report (“Unlocking new opportunities: jobs in green and healthy transport”) [13] estimated that an additional 76,600 jobs could be created if the selected cities invested in cycling to reach the same modal share as Copenhagen. The first report also concluded that the benefits of cycling to health could prevent about 10,000 deaths each year in the cities included in the study. For the pan-European region as a whole, these figures are likely to be underestimates, as they correspond to only one city per country and to only a small proportion of the jobs that could contribute to making transport greener, safer and more efficient. The estimates include cycling-related employment in retail, wholesale, and design, so jobs associated with administration, construction, and tourism, for example, were excluded. Consequently, it was not surprising that the information collated for the more recent report led to a higher estimate of the number of jobs associated with cycling [11]. With the new evidence that emerged from the data collection exercise for the second report, it was estimated that an additional 435,000 cycling-related jobs might be created if the same 56 major cities had the same cycling modal share as Copenhagen [11] (see Table 2).

**Figure 2:**
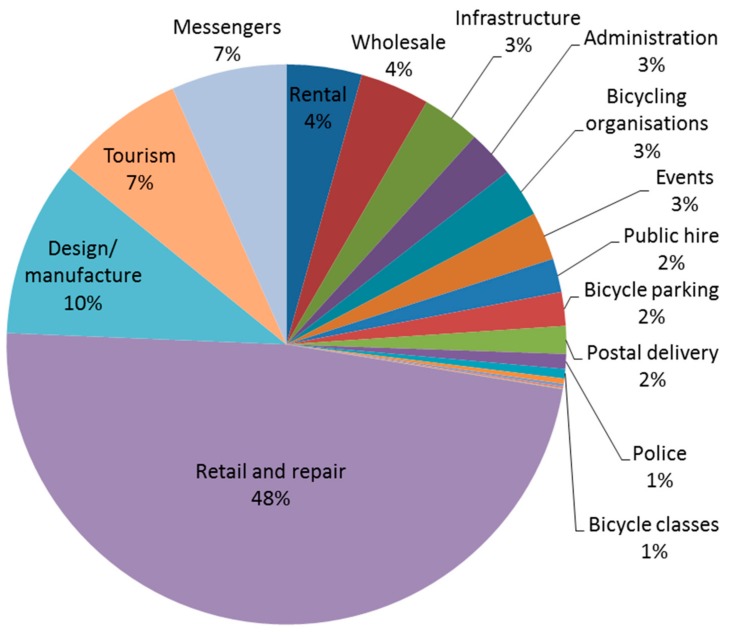
Cycling jobs identified by category in 34 cities. Source: WHO Regional Office for Europe; United Nations Economic Commission for Europe. Riding towards the green economy: cycling and green jobs; WHO Regional Office for Europe: Copenhagen, Denmark, 2016. Available online: http://www.euro.who.int/__data/assets/pdf_file/0017/311471/Cycling-and-green-jobs.pdf (accessed on 3 November 2017) [11]. Notes: Data on jobs in Copenhagen, Vienna, and Münster are not included in this figure because the study could not access detailed information from these cities. Data was obtained primarily from the submitted estimates for this report; except in the case of Brussels, in which data was taken from TML and Pro Vélo [17].

Figure 2 illustrates the variety of types of cycling job that exist, according to the information collected for the more recent report [11]. Beyond the obvious jobs in design and retail, many cities have several other services using bicycles: for example, bicycle messengers, bicycle taxis, and bicycle logistics (use of bicycles for delivering merchandise in cities). As yet, the number of jobs in bicycle taxis and bicycle logistics are relatively small in number, and are often present in cities with a higher level of cycling, but it demonstrates once more the potential for investment in cycling to increase the number and range of jobs.

### 3.1. Direct Job Creation

**Figure 3:**
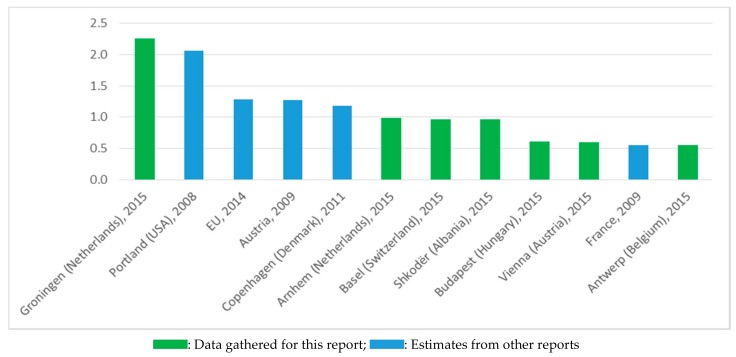
Locations with some of the highest number of direct cycling-related jobs per 1000 residents [11]. Source: WHO Regional Office for Europe, United Nations Economic Commission for Europe, “Riding towards the green economy: cycling and green jobs”, 2017. Copenhagen: WHO EURO [11].

Figure 3 presents the cities and regions with the largest number of jobs associated with cycling from both this report [11] and other reports that were reviewed as part of the study [18,19,20,21,22]. These jobs were largely direct jobs, as induced jobs were not included and indirect jobs were only partially included in these estimates. It should also be remembered that the estimates from other reports used different methodologies, such as the figure for Copenhagen covered a narrower range of jobs than the estimates obtained for the recent report undertaken within the context of THE PEP [11].

An interesting observation is for the US city of Portland. This is the only city for which we have more than one estimate of the number of cycling jobs from other sources. The number of jobs indicated in Figure 3 for Portland is the high estimate in a second study for the city; the first, from 2006, had a lower number of jobs associated with a lower level of cycling. While it is perhaps not surprising that the quantity of jobs associated with cycling increases as cycling increases, it is still worth noting that evidence from Portland demonstrates this. Neither of the reports from Portland translate cycling levels to a modal share figure, so it is not possible to identify the relationship between jobs per 1000 people and cycling modal share for Portland over time. Instead, the reports present figures relating to bicycle counts over bridges, which increased by 39% between 2006 and 2008, and the use of the cycle lane network, which only increased by 2% in the same period (although this increase was not considered to reflect the increase in cycling in the city as a whole) [11]. This compares with an increase in the number of jobs of around 43% (taking the middle of the range presented in each case), which perhaps suggests that the number of jobs associated with cycling increases faster than the level of cycling (and so their relationship is not linear). More research would be needed to confirm this conclusion. 

The fact that the number of cycling jobs estimated directly by six major cities for the second report [11] was on average more than 150% higher than that estimated for our first report [13] also suggests that the relationship between the number of jobs and cycling levels, in these cases presented in terms of modal share, is not linear. This is due to the fact that assuming a linear relationship in the first report under-estimated the number of cycling jobs in these six cities, according to the estimates provided by the cities themselves. This suggests that other factors, including improved facilities, infrastructure, and culture for cycling, might also have an impact on the number of cycling jobs.

### 3.2. Indirect and Induced Job Creation 

Furthermore, comparing the findings from other national studies to the estimates for cities in the recent report, it appears that there is great potential for cycling related jobs outside cities. Other studies that have estimated the number of jobs at a national or European level have suggested that cycling tourism provides a significant share of the total number of jobs associated with cycling. Indeed, the share of cycling-related jobs related to the tourism in Austria [20] and France [22], for instance, are estimated to be 70% and 47% of the total number of cycling related jobs, respectively. On the other hand, only around 7% of the cycling jobs identified in the latest study were associated with cycling tourism. 

Moreover, when the economic domino effect is triggered, more jobs are created throughout the consumption and production chain. In fact, the amount of induced jobs generated by the increased global level of spending in the economy as a consequence of the higher level of employment in cycling is potentially meaningful. One study on the Austrian Cycling Master Plan [23] for the period of 2015–2025 estimated that the amount of induced jobs, especially in the tourism industry, may well account for around 40% of the overall number of cycling-related jobs.

## 4. Discussion

The summarized report and THE PEP partnership on jobs in green and healthy transport aim to communicate to public authorities the potential benefits of cycling in creating jobs and delivering a greener and healthier transport system. The results presented above demonstrate both the potential number of jobs that might be created from increasing the modal share of cycling and the range of these jobs. This underlines the economic, as well as environmental and health benefits of cycling. The inclusion of such evidence in the appraisal and planning of municipal transport policies would enable a more comprehensive assessment of the benefits of cycling. It would also allow cycling to deliver a greener and healthier transport system from local to national levels. 

Besides its health promotion mechanisms, cycling can also reduce premature mortality. When using the WHO’s HEAT tool [24], it was calculated that if the 56 selected cities would increase the modal share of cycling to Copenhagen’s, around 10,000 premature deaths per year would be avoided [13]. Cycling at the WHO Guidelines recommended level for physical activity (ca. 150 min per week of moderate-intensity physical activity for adults) reduces all-cause mortality by around 10% [25]. 

In view of all the benefits of cycling, its promotion provides an excellent way to move towards achieving many of the Sustainable Development Goals (SDGs), including numbers 3 and 11 on ensuring healthy lives and promoting well-being for all at all ages and making cities and human settlements inclusive, safe, resilient, and sustainable. In addition, the promotion of cycling would contribute to SDG 12, ensuring sustainable consumption and production patterns, and could be part of a set of actions to implement SDG 13, taking urgent action to combat climate change and its impacts. Moreover, supporting cycling and stimulating job creation relates directly to the WHO European policy for health and well-being Health 2020’s priority [26] related to creating supportive environments and resilient communities.

### 4.1. How Local Governments Can Support Job Creation through Cycling

In order to foster job creation, local authorities need to be proactive in promoting cycling:

- First, municipal authorities should facilitate and support the expansion of cycling as a central part of a multifaceted transport scheme. Cycling should be fully incorporated into a multimodal city transport policy integrated into a broader active mobility policy. At the same time, any obstacles to implementing the policy should be assessed and monitored.
- Second, improving the capacity of cities to create jobs in cycling involves analyzing the existing job market and understanding the processes required to develop cycling businesses. The collected evidence can then be included in the preparation and elaboration of municipal policies to increase the share of cycling in the urban transport scheme.
- Third, coordinating a cycling plan for infrastructure and economic conditions for business development in tourism with regional and national authorities might have a significant impact on the overall number of induced jobs.
- Fourth, disincentive policies on the use of private cars in urban areas, especially in the city center, would trigger a higher use of bicycles if cycling infrastructure, integration of cycling with public transport and cycling-friendly culture reached a minimal level.

Finally, local governments have to be aware that jobs might migrate from certain sectors related to the current modes of transportation to cycling related jobs. Given the nature of the jobs created, many of these additional jobs will be local, rather than in remote car factories, which is positive for the local economy. As with all new job creation, national and local authorities will have to be vigilant and take action when necessary, to ensure that the jobs created, many of which will be in the service sector, are “decent work” of good quality and that the employees have sufficient protection under the law. According to the International Labour Organization (ILO) “decent work sums up the aspirations of people in their working lives. It involves opportunities for work that is productive and delivers a fair income, security in the workplace and social protection for families, better prospects for personal development and social integration, freedom for people to express their concerns, organize and participate in the decisions that affect their lives and equality of opportunity and treatment for all women and men” [27].

### 4.2. Impacts on Other Sectors

Increasing the modal share of green and healthy transport may lead to some job losses in certain sectors, such as in car manufacture and servicing. The reviewed studies, and evidence collated, did not attempt to estimate any potential job reduction in other sectors; further investigation would be necessary in order to better understand the market dynamics in related sectors. The Austrian economist Joseph Schumpeter calls this process creative destruction: the “process of industrial mutation that incessantly revolutionizes the economic structure from within, incessantly destroying the old one, incessantly creating a new one” [28]. According to Schumpeter’s innovation theory, rather than acting as a supplement, the cycling economy might partly substitute the private car economy. Transformations in the modal share would not only affect the use of public spaces and city planning, but may also have a larger societal and economic impact in terms of consumption and production. In general, increasing the modal share of cycling would result in a wide range of benefits for public health and the environment [6,8,9], in addition to a potential increase in the number of jobs. There are risks that other sectors might have the number of jobs reduced due to the modal share transition, but the net, long-term impact is more difficult to identify, as it is also dependent on developments within the transport industry (such as automation and delocalization of production) which are independent from trends in cycling. 

Further investigation on how the jobs in different sectors evolved in relation to demographic changes and in the modal share of cycling, for example in Amsterdam or Copenhagen, would be necessary in order to better understand the real job creating potential and the proportion between new jobs and the ones substituting old ones during the modal share transition.

## 5. Future Research Aspects

The study was based on data provided by the administration of each selected city. It was clear from some of the responses that the data were collected in different ways and using varying definitions, in spite of the development of a common data collection framework. There was also the potential that city authorities might have provided biased estimates in order to promote a more positive image about their cities than justified by the evidence. Overcoming these methodological challenges would require more resources in order to ensure that data were collected in a consistent, coherent, and objective fashion in order to deliver results in which we could have more confidence. Disaggregating the data typically collected by city authorities would help identify the precise contribution of cycling to various statistics, especially those relating to employment.

However, as the methodology used was only a simple extrapolation, it raises the question of the extent to which the job estimates provided are representative and achievable for the rest of Europe remains open. Copenhagen has spent many years—perhaps decades—reaching the levels of cycling that it currently has as a result of a long-running dedicated policy in this area. In this respect, although it offers an inspiring example of what is possible to achieve, it is clearly not representative of the vast majority of other cities. Other cities in the pan-European region are far behind Copenhagen in this respect (as is clear from Table 2), and so some would probably take decades to reach the levels of cycling seen in Copenhagen. Consequently, even if all of the cities were able to follow exactly the same trajectory as Copenhagen, the associated number of cycling jobs would be delivered at different times in different cities, as a result of their varying starting points. 

The extent to which all cities would be able to follow the trajectory undertaken by Copenhagen is also questionable. Each country—let alone city—has different policy and planning cultures, so many may take even longer to increase cycling to anywhere near the levels of Copenhagen. Consequently, the job increases estimated for each city may not be achievable in practice. Given these issues, it is clearly therefore challenging to set confidence intervals for the potential increase in the number of jobs. Consequently, in order to guide policy within a city, objectives of perhaps doubling cycling in a specified time period, for example 10 years, might be a more appropriate target and so be a better basis on which to estimate the potential increase in the number of jobs for an individual city. 

The study suggests also that tourism could be an important source of employment in cycling related jobs. The low values found in this study [11] contrast to the high proportion of cycling jobs estimated in studies that covered wider geographical areas [20,22]. Thus, further investigation of the distribution of jobs and the mechanisms involved in their creation would provide valuable evidence for public authorities to better focus on creating the conditions that enable job generation. In fact, another issue that was mentioned in the studies that deserves further discussion and investigation are the social and economic differences between the types of jobs created and those replaced. Once the modal share of transportation changes the respective jobs are partially substituted. As the new jobs created are expected to be mostly in the services sector, their status, wage, and contractual conditions might be of a different level that the ones they replace. 

An important finding from the report was that the relationship between the amount of cycling-related employment, the size of the city, and its modal share for cycling is multifaceted. Therefore, more consistent data collection would facilitate further research that could be used to develop a method for predicting how cycling jobs might increase in response to higher modal shares. A transition to a new stage with a higher modal share and employment in cycling is probably not linear as assumed, since certain thresholds will need to be reached so that some services become viable and needed. An additional area of research would be to understand better the nature and type of potential job losses in other sectors resulting from an increase in the number of cycling jobs and compare these in terms of quality, security, and potential wages to the jobs created.

## 6. Conclusions

Evidence has shown that developing cycling in major European cities not only has a significant positive impact on the environment and health of urban citizens but it can also lead to the creation of cycling-related jobs in cities and surrounding areas. As the modal share of cycling increases, there is evidence that the number of jobs associated with cycling also increases and that these jobs become more varied.

As an important component of active mobility, cycling not only promotes environment protection, but when it comes to health, it can also reduce mortality. Hence, investments in cycling should be recognized for their economic benefits, as well as their health and environmental benefits.

## Figures and Tables

**Table 1:** Summary of the relationship between physical activity and health.

Health Topic	Evidence of the Effect of Physical Activity	Strength of Evidence
Overall death rate	Approximately 30% risk reduction for the most active compared with the least active	Strong
Cardiovascular health	20% to 35% lower risk of cardiovascular disease, coronary heart disease and stroke	Strong
Metabolic health	30% to 40% lower risk of type 2 diabetes in at least moderately active people compared with those who are sedentary	Strong
Musculo-skeletal health	36% to 68% risk reduction of hip fracture at the highest level of physical activity	Moderate
Falls	Older adults who participate in regular physical activity have an approximately 30% lower risk of falls	Strong
Cancer	Approximately 30% lower risk of colon cancer and 20% lower risk of breast cancer for adults participating in daily physical activity	Strong
Mental health	Approximately 20% to 30% lower risk for depression and dementia for adults participating in daily physical activity.	Strong

Source: Department of Health, 2011 Start Active, Stay Active: A report on physical activity from the four home countries’. London: DH [10].

**Table 2:** Selected major cities, including population, cycling modal shares, estimated current cycling-related jobs and potential [additional] cycling-related jobs [11].

Country	City	Population (Millions)	Cycling Modal Share	Estimated Existing Cycling-Related Jobs	Estimated Potential [Additional] Cycling-Related Jobs
Albania	Tirana	0.80	3% ^a^	150	1150
Andorra	Andorra-La-Vella	0.022	3% ^a^	17	133
Armenia	Yerevan	1.12	3% ^a^	875	6709
Austria	Vienna	1.77	7%	1058	2872
Azerbaijan	Baku	2.12	3% ^a^	1655	12,691
Belarus	Minsk	1.89	0%	98	12,645
Belgium	Brussels	1.08	4%	230	1479
Bosnia and Herzegovina	Sarajevo	0.31	3% ^a^	238	1825
Bulgaria	Sofia	1.17	1%	304	7605
Canada	Ottawa	1.24	2%	644	7732
Croatia	Zagreb	0.79	5%	1031	4329
Cyprus	Nicosia	0.055	3% ^a^	43	329
Czechia	Prague	1.24	1%	323	8071
Denmark	Copenhagen	0.55	26%	3712	0
Estonia	Tallinn	0.40	4%	417	2294
Finland	Helsinki	0.60	7%	1084	2941
France	Paris	2.23	3%	1743	13,360
Georgia	Tbilisi	1.17	3% ^a^	911	6982
Germany	Berlin	3.50	13%	11,836	11,836
Greece	Athens	2.48	2%	905	10,860
Hungary	Budapest	1.74	2%	1049	10,809
Iceland	Reykjavik	0.12	3% ^a^	92	706
Ireland	Dublin	0.53	3%	412	3155
Israel	Tel Aviv	0.40	9%	947	1788
Italy	Rome	2.76	0%	287	18,380
Kazakhstan	Astana	0.66	1%	172	4301
Kyrgyzstan	Bishkek	0.89	3% ^a^	694	5320
Latvia	Riga	0.65	3% ^a^	507	3890
Liechtenstein	Vaduz	0.0052	3% ^a^	4	31
Lithuania	Vilnius	0.55	1%	144	3588
Luxembourg	Luxembourg-Ville	0.01	3% ^a^	78	597
Malta	Valletta	0.0062	3% ^a^	5	37
Monaco	Monaco City	0.036	3% ^a^	28	217
Montenegro	Podgorica	0.18	3% ^a^	141	1081
Netherlands	Amsterdam	1.07	33%	9170	0 ^b^
Norway	Oslo	0.60	5%	779	3272
Poland	Warsaw	1.71	5%	2134	9426
Portugal	Lisbon	0.47	1%	123	3086
Republic of Moldova	Chisinau	0.79	3% ^a^	616	4721
Romania	Bucharest	1.94	1%	504	12,593
Russian Federation	Moscow	11.54	3% ^a^	9002	69,015
San Marino	San Marino	0.0045	3% ^a^	3	27
Serbia	Belgrade	1.64	1%	426	10,657
Slovakia	Bratislava	0.41	3% ^a^	321	2463
Slovenia	Ljubljana	0.28	10%	110	176
Spain	Madrid	3.27	1%	849	21,223
Sweden	Stockholm	0.86	1%	225	5618
Switzerland	Berne	0.12	11%	356	485
Tajikistan	Dushanbe	0.70	1% ^a^	183	4576
The former Yugoslav Republic of Macedonia	Skopje	0.32	3% ^a^	247	1895
Turkey	Ankara	4.89	3% ^a^	3815	29,248
Turkmenistan	Ashgabat	0.64	3% ^a^	497	3809
Ukraine	Kyiv	2.77	1% ^a^	721	18,023
United Kingdom	London	7.83	3%	6104	46,799
United States of America	Washington, DC	0.62	3%	482	3696
Uzbekistan	Tashkent	2.30	1% ^a^	597	14,927
Total					435,480

^a^The actual modal share for cycling for the marked cities could not be identified. In those cases, low cycling modal shares of 3% or 1% were presumed; ^b^ In the case of Amsterdam no supplementary cycling-related jobs are projected, since the level of cycling is higher than Copenhagen’s Source: WHO Regional Office for Europe; United Nations Economic Commission for Europe. “Riding towards the green economy: cycling and green jobs”; WHO Regional Office for Europe: Copenhagen, Denmark, 2016. Available online: http://www.euro.who.int/__data/assets/pdf_file/0017/311471/Cycling-and-green-jobs.pdf (accessed on 3 November 2017) [11].
